# Effects of n-3 polyunsaturated fatty acids high fat diet intervention on the synthesis of hepatic high-density lipoprotein cholesterol in obesity-insulin resistance rats

**DOI:** 10.1186/s12944-016-0250-3

**Published:** 2016-04-22

**Authors:** Xianxing Xie, Tao Zhang, Shuang Zhao, Wei Li, Lanzhi Ma, Ming Ding, Yuan Liu

**Affiliations:** Laboratory Animal Center of the Academy of Military Medical Sciences, Beijing, 100071 China

**Keywords:** n-3 polyunsaturated fatty acids, Serum lipids, Liver, High density lipoprotein cholesterol, Insulin resistance

## Abstract

**Background:**

n-3 polyunsaturated fatty acids (PUFA) have previously been demonstrated in association with a reduced risk of chronic diseases, including insulin resistance, cancer and cardiovascular disease. In the present study, we analyzed the effects of n-3 PUFA-rich perilla oil (PO) and fish oil (FO) high fat diet intervention against the synthesis of hepatic high-density lipoprotein cholesterol (HDL-c) in obesity-insulin resistance model rats.

**Methods:**

In the modeling period, the male SD rats were randomly divided into 2 groups. The rats in the high fat (HF) group were given a high fat pure diet containing 20.62 % lard. In the intervention period, the model rats were intervened with purified high-fat diets rich in PO or FO, containing same energy content with high fat pure diet in HF. After the intervention, the protein and mRNA expressions status of the key genes involved in synthesis of hepatic HDL-c were measured for further analytic comparison.

**Results:**

The obesity-insulin resistance model rats were characterized by surprisingly high levels of serum triglyceride (TG) and increased body weight (*P* < 0.05, each). After the intervention, there were no apparent changes in the serum HDL-c and total cholesterol (TCH). In addition, the FO could up-regulate the hepatic adenosine triphosphate (ATP) binding cassette transporter A1 (ABCA1) mRNA (*P* < 0.01) and protein expressions, as well as increase the level of serum apolipoprotein A-1 (apoA-1) (*P* < 0.0001), and elevate the hepatic apoA-1mRNA expression (*P* < 0.01). Different from FO, the PO specifically elevated the hepatic ABCA1mRNA expression (*P* < 0.01).

**Conclusions:**

The FO high fat diets promoted the synthesis of HDL-c in the obesity-insulin resistance rats.

## Background

Insulin resistance (IR), characterized by the impairment of insulin action, is an important metabolic alteration which is frequently associated with obesity, and appears to be the primary mediator of metabolic syndrome (MetS) [1]. Extensive research results have revealed that obesity can lead to IR through endocrine mechanism, oxidative stress, decreased fat oxidation capacity, and adipose tissue dysfunction [2, 3]. Obesity represents the major risk factor in the development of insulin resistance during childhood and adolescence [4]. With the increase in the consumption of western diets with high fat content, studies have confirmed that long-term intake of excessive amounts of fat, especially saturated fat, is a key factor leading to insulin resistance and obesity [5–7]. The obesity-insulin resistance is often accompanied blood lipids disorder and hepatic infiltration of excess free fatty acids, which are high risk indicators for the development of MetS, also called “syndrome X” can be defined as a cluster of abnormalities [8–10]. Furthermore, the conditions most commonly associated with low HDL-c, the insulin-resistant metabolic syndrome, in association with the increased catabolism of HDL and the mechanism, are poorly understood [11]. Aside from the antioxidant, anti-inflammatory and antithrombotic effects of HDL, its cardioprotective effects have been largely attributed to its role in the reverse cholesterol transport (RCT) pathway, in which excess cholesterol in the peripheral tissues is returned to the liver for utilization or elimination [12]. Although HDL promotes RCT from the extrahepatic tissues to liver, the liver itself is a major source of lipidation of nascent HDL. It is of great importance to control not only the blood glucose but also the lipid profile to prevent the onset of MetS [13]. Therefore, it may be useful to seek therapeutic drugs and therapies to ameliorate blood lipid disorder by regulating hepatic HDL metabolic pathways in insulin resistance models.

The US Food&Drug Administration (FDA) has granted a qualified health claim for dietary n-3 PUFA supplements: ‘Consumption of ω-3 fatty acids may reduce the risk of coronary heart disease’ [14]. n-3 PUFA, such as a-linolenic acid[18:3(n-3), ALA], eicosapentaenoic acid[20:5(n-3), EPA] and docosahexaenoic acid[22:6(n-3), DHA], have demonstrated a wide range of health-related benefits, including improving heart disease related outcomes, decreasing tumour growth and metastasis, and favourably modifying insulin sensitivity [15–19]. EPA and DHA, which are mainly ingested from fatty fish and marine animals, have been studied extensively, whereas ALA, found in vegetable foods, lacks substantive evidence for a biological role. The beneficial effects are influenced by the types and quantities of n-3 PUFA, but the mechanisms are less clear.

Currently, the regulation of n-3 PUFA high fat diets on the synthesis of hepatic HDL-c in vivo has yet to be determined. The present study mainly explores the effects of n-3 PUFA-rich PO and FO high fat diets on the serum lipids of obesity-insulin resistance rats, as well as the expression of the key genes involved in synthesis of hepatic HDL-c.

## Results

### Diet intake and body weight in the modeling period

As illustrated in Table 1, the diet intake of the HF rats was shown to be significantly lower than that of the normal control (NC) group (*P* < 0.0001). However, the energy intake and fat-energy intake were significantly higher than those of the NC group (*P* < 0.0001, each). At the end of the animal modeling period, the body weights of the HF rats were significantly higher than those of the NC group (*P* = 0.0186).

**Table 1:** Diet intake and body weight in the model construction period

Item	NC	HF	*P*
Diet consumption(g/d)	21.57±2.13	19.17±2.16	<0.0001
Energy intake(kJ/d)	345.25±33.51	373.95±41.87	<0.0001
Fat energy intake(kJ/d)	35.60±3.45	171.01±19.15	<0.0001
Weight(g)	587.75±48.77	637.43±69.28	0.0186

### Insulin sensitivity

**Fig. 1:**
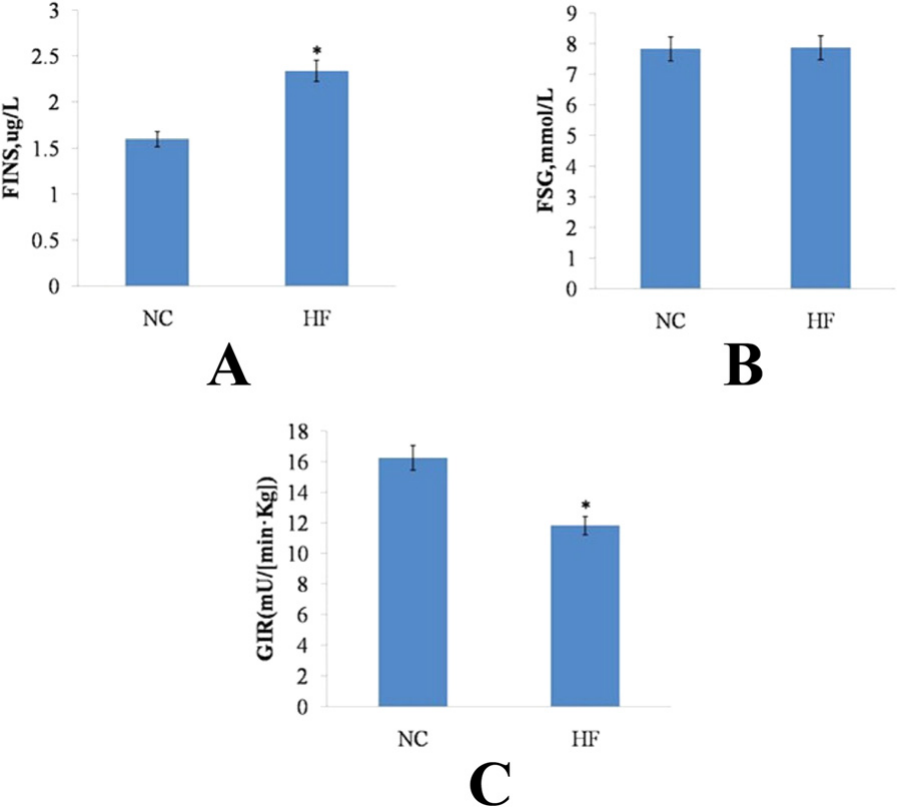
Insulin sensitivity. **a**, fasting insulin; **b**, fasting serum glucose; **c**, glucose infusion rate. Data are mean ± standard deviation (SD), in (**a**) and (**b**) *n *= 8 rats per diet, in (**c**) *n* = 4 rats per group. Significance during the HF compared to NC is indicated by * *P* < 0.05

Figure 1a illustrates the fact that the levels of fasting insulin (FINS) in HF were higher than those in NC (*P* < 0.05), and that there was no statistical difference in fasting serum glucose (FSG), as shown in Fig. 1b. Figure 1c illustrates the fact that the HF rats exhibited insulin resistance, due to the fact that the glucose infusion rate (GIR) in the HF was significantly lower than that of the NC in the euglycemic-hyperinsulinemic clamp study (*P* = 0.0086).

### Serum parameters of obesity-insulin resistance rats

**Fig. 2:**
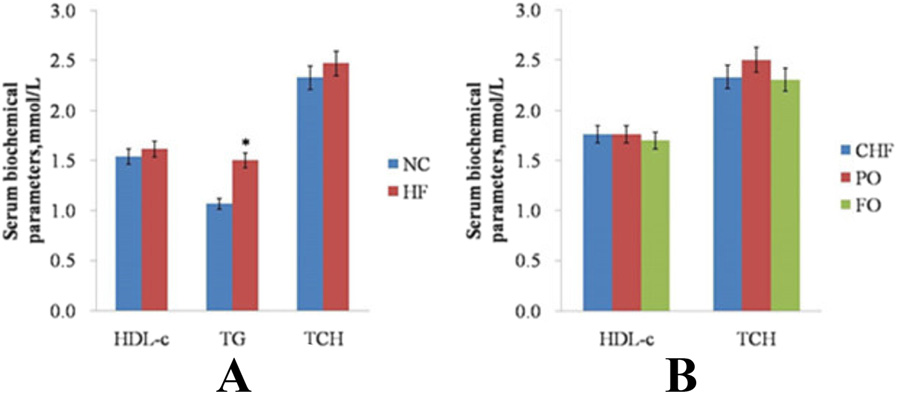
Serum biochemical parameters. (**a**) Serum biochemical parameters of obesity-insulin resistance rats. (**b**), Serum biochemical parameters of the rats after the intervention. Data are mean ± standard deviation (SD), *n* = 12 Rats per diet. “*” (*p* < 0.05) means it is significantly different with the NC or CHF group

Following the 14 h fasting period, the serum TG of the rats fed with HF diet was found to be remarkably higher than those fed with NC diet (*P* < 0.05), and there was no significant difference in TCH and HDL-c, as shown in Fig. 2a.

### Serum parameters of the rats after the intervention

After an intervention, there were no apparent changes in the serum HDL-c and TCH, as illustrated in Fig. 2b.

### Analysis of the differential expressions of hepatic ABCA1 and apoA-1 by real-time quantitative PCR

**Fig. 3:**
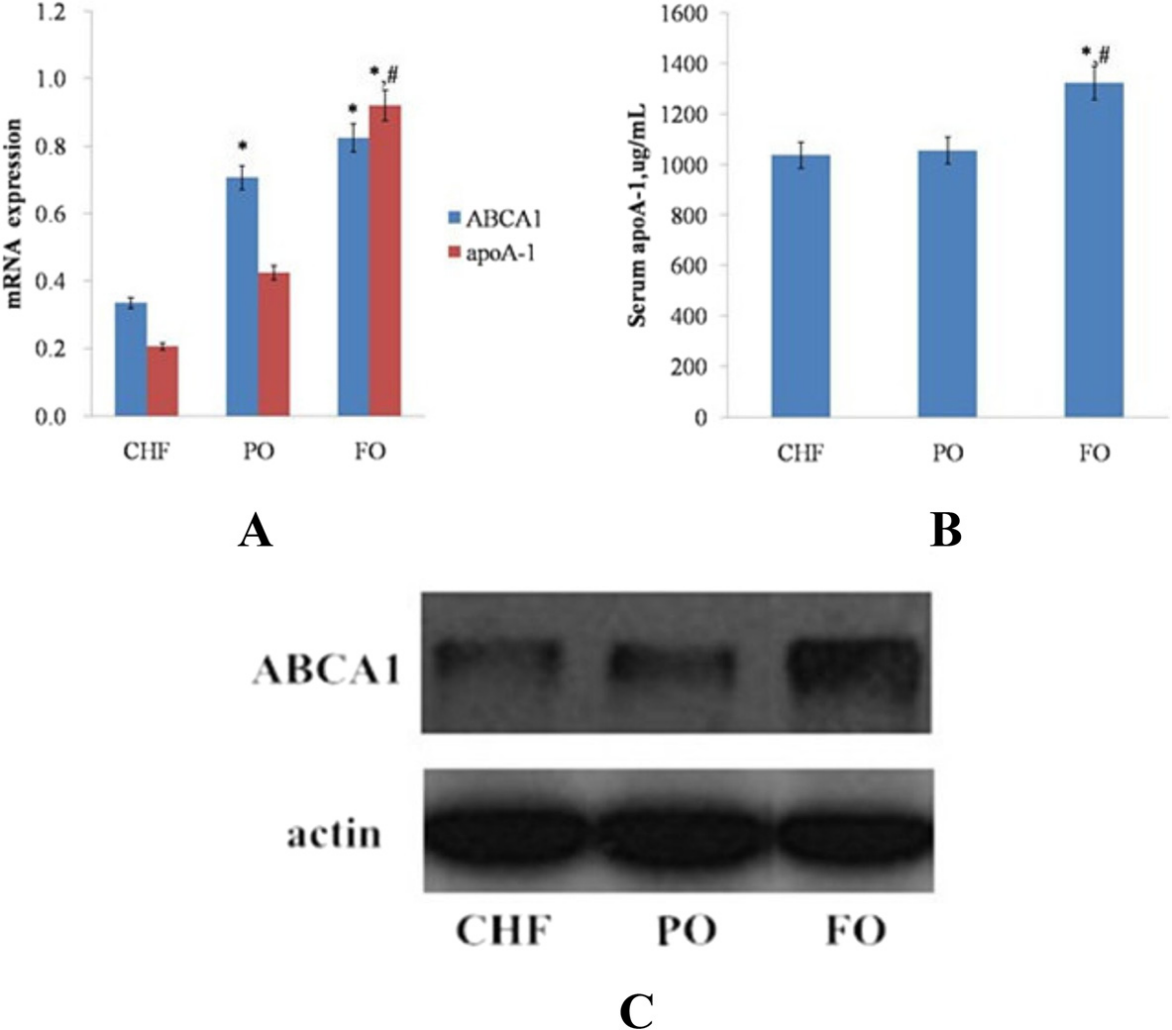
The effect of n-3 PUFA high fat diets on mRNA and protein expression. (**a**), Relative gene expression levels of hepatic ABCA1 and apoA-1; (**b**), Serum apoA-1; (**c**), Protein expressions of hepatic ABCA1. Data are mean ± standard deviation (SD), *n* = 6 Rats per diet. “*” (*p* < 0.05) means it is significantly different with the CHF group and “#” (*p* < 0.05) means it is significantly different with the PO group

The real-time qPCR results showed that, after n-3 PUFA intervention, the expression levels of hepatic ABCA1 mRNA were remarkably increased (*P* < 0.01), and there was no statistical difference between the PO and FO. In contrast, the expression levels of hepatic apoA-1 mRNA in FO were significantly higher than those in the continuous high fat (CHF) and PO, as shown in Fig. 3a (*P* < 0.01, each).

### Detection of serum apoA-1 by ELISA

The study results shown in Fig. 3b illustrated the fact that the serum apoA-1 in the FO was significantly higher than that of the CHF and PO (*P* < 0.0001, each), and that there was no statistical change between the CHF and PO.

### Detection of hepatic ABCA1 protein levels by Western blotting

In order to further understand the effects of n-3 PUFA high fat diet on obesity-insulin resistance model rats, we investigated the effects of n-3 PUFA on key protein expressions for the synthesis of hepatic HDL-c. After the FO intervention, the expression levels of the ABCA1 protein were dramatically increased, but there was no apparent change in the PO intervention group, as shown in Fig. 3c.

## Discussion

In the present study, we investigated the modulatory role of dietary n-3 PUFA high-fat diets against the synthesis of hepatic HDL-c in obesity-insulin resistance model rats. For the first time, it was shown that n-3 PUFA may promote the synthesis of HDL by up-regulating the expression of key gene ABCA1. Although they share the same effects on serum HDL-c, the FO and PO exerted different influences on the hepatic HDL-c biosynthesis in the obesity-insulin resistance rats.

High insulin levels resulting from an insulin resistance lead to blood lipid abnormalities. In animal models, our results illustrated that a high-fat diet induced insulin resistance in rats with increasing serum TG. As we all know, in the presence of insulin resistance, HDL-c levels often are reduced [20]. But in modeling and intervention period, there are no apparent changes in the serum HDL-c and TCH. Several epidemiological studies have shown that HDL-c concentrations tend to increase when the diet is rich in n-3 PUFA, Morvan et al. firstly report that n-3 PUFA could decrease serum HDL-c level [21, 22]. Since the anti-atherogenic property of HDL is not simply determined by plasma HDL-c concentrations but depends also on the properties of HDL [23], the effects of n-3 PUFA on HDL functionality RCT need to be investigated. n-3 PUFA has been shown to accelerate the last step of RCT in mice, but the activity of the key protein for the synthesis of HDL-c was an important determinant for the beginning of RCT [22, 24]. Nutrient deprivation can lead to the alteration both *in vitro* and *in vivo,* such as cellular macromolecular composition change, apoptosis of endothelial cell, and diminishment of insulin signaling in the activation of neuronal autophagy [25, 26]. So we keep diet energy equal between modeling and intervention period. Therefore, in this study we explored the effects of the different high fat diets of n-3 PUFA on the expression of the key protein for the synthesis of HDL-c.

The initial step in HDL formation involves the synthesis and secretion of apoA-1 by the liver cells, and, to a lesser extent, by the intestinal cells [27]. As the main apolipoprotein of HDL-c, apoA-1 is secreted in a lipid-poor form, and accounts for approximately 70 % of the lipoprotein [28]. ApoA-1 interacted with ABCA1 mediating the efflux of cholesterol and phospholipids, and participated in RCT. ApoA-1 overexpression promotes macrophage-specific RCT in vivo [29]. Presently, the available data are insufficient to assess whether the supplementation of HF diets with n-3 PUFA, carried either by PO or FO, can make any difference in the apoA-1 metabolic outcomes, with the aim of preventing or treating metabolic disorders. In our study, the serum apoA-1 and hepatic apoA-1 mRNA expressions were significantly increased in the obesity-insulin resistance rats after the FO intervention, and there was no statistical change between the CHF and PO. This indicated that an FO high-fat diet may promote RCT, and potentially protect the vascular fatty degeneration in the obesity-insulin resistance rats. Different from our high-fat diet results, the usual diets enriched with FO decreased the apoB-100/apoA-1 ratio without a significant influence on serum apoA-1 [30]. Therefore, the conditions of high-fat diets may affect the physiological functions of FO. Given that there were no significant changes on the apoA-1 expression, the PO high-fat diets may not have apoA-1 inducing beneficial effects. Moreover, the FO compared with PO exhibited stronger beneficial effects in increasing the level of serum apoA-1 and up-regulating hepatic apoA-1 mRNA expression. This may provide valuable evidence for further investigation regarding the different benefits of n-3 PUFA.

The ABCA1 is a membrane protein localized to the non-lipid raft area of the plasma membrane, and mediates the efflux of phospholipids and cholesterol of the cells. It facilitates the translocation of phospholipids to free apoA-1 in the plasma membrane, forms pre-β-HDL, and initiates RCT [31]. Therefore, the ABCA1 plays a rate limiting role in the first step of the synthesis of the HDL and RCT processes [29]. A high expression of ABCA1 in the macrophages accelerated the HDL synthesis, enhanced the level of serum HDL-c, and accelerated the RCT [32]. In in vitro studies, the ALA cannot change the expression of the ABCA1 in foam cells, and EPA is known to down-regulate ABCA1 in macrophages, which is mostly due to their different effects on Liver X receptors (LXRs) [33, 34].There is no direct result of the n-3 PUFA-rich PO and FO high-fat diets on the expression of hepatic ABCA1. In the present study, the hepatic ABCA1 mRNA expressions in the n-3 PUFA intervention groups were significantly higher than those of the CHF. Therefore, it appears that n-3 PUFA high fat diets may enhance the hepatic ABCA1-mediated efflux of cholesterol and phospholipids to apoA-1 containing nascent HDL particles in obesity-insulin resistance rats. Consistent with our findings, the high dietary ratio of n-6/n-3 PUFA can significantly increase the expression of ABCA1 essential for the metabolism of HDL and RCT [35]. n-3 PUFA may exhibit different functions in in vivo and in vitro studies. In addition, the FO also exhibited stronger beneficial effects than PO in up-regulating hepatic ABCA1 protein expressions in the obesity-insulin resistance rats. Although they share the same effect on serum HDL-c, the FO and PO high-fat diets exerted different influences on hepatic HDL-c biosynthesis in the obesity-insulin resistance rats.

## Conclusions

The n-3 PUFA-rich PO and FO high fat diets were found to upregulate the hepatic ABCA1 mRNA expression in the obesity-insulin resistance rats. Furthermore, although sharing the same effect on serum HDL-c, FO and PO high fat diets exerted differently influence on hepatic HDL-c biosynthesis in the obesity-insulin resistance rats. In summary, FO high-fat diets may promote the synthesis of HDL-c in obesity-insulin resistance rats.

## Methods

### Animals

In this study, 48 specific pathogen-free (SPF) male Sprague–Dawley (SD) rats were purchased from the Laboratory Animal Center of the Academy of Military Medical Sciences (Beijing, China), and the certificate number of SCXK-(Army) 2007–004. The rats were housed in micro-isolator cages with a light/dark cycle of 12 h (6: 00 to 18: 00) and given free access to diet and water. The temperature was controlled at 23 ± 1 °C, and the relative humidity was 40 ± 5 %. All animal experiments were performed in accordance with the guide of the Institutional Animal Care and Use Committee of Laboratory Animal Center.

### Diets

The purified daily diets were formulated according to the production of Test diet 58Y2 and 58 V8, which were mainly composed of casein, maltodextrin, corn starch, methionine, fat, cellulose, minerals, sucrose, and vitamins. The NC group diet was added 2.4 % soybean oil and 1.9 % of lard oil, with an energy content of 15.7 kJ/g, of which the protein-energy, carbohydrate-energy, and fat-energy ratios were 20.2 %, 69.49 %, 10.31 %, respectively. Along with the addition of the 2.88 % soybean oil, HF/CHF, PO intervention, and FO intervention groups were also added, which included 20.62 % lard, 4.12 % perilla oil + 16.50 % lard, and 4.12 % fish oil + 16.50 % lard, containing the same energy content of 19.35 kJ/g, of which the protein-energy, carbohydrate-energy, and fat-energy ratios were 19.67 %, 34.59 %, 45.73 %, respectively. The fatty acid compositions of PO and FO are listed in Table 2.

**Table 2:** Types and levels of dietary fatty acids

Fatty acids	Fatty acids in oil ,g/100 g	
	PO	FO
C18:3	60.64	5.39
C20:5	0	19.84
C22:6	0	14.00
Total	60.64	39.23

### Groups

The 48 SPF male SD rats were randomly divided into 2 groups: 12 rats in the NC group, and 36 rats in the HF group for modeling obesity-insulin resistance rats. After the HF rats developed insulin resistance, they were randomly divided into 3 groups: CHF group, PO intervention group, and FO intervention group. After the intervention at 4 weeks, animals were euthanasia, and the liver tissue samples were collected for cryopreservation with liquid nitrogen.

### Serum parameters

Before collecting blood, fasting was conducted on the rats for a 14 h period (18:00 to 8:00) to ensure free access to water. Then, centrifugation was carried out in order to collect supernatant at 1000 × g for 10 min at 4 °C and stored at −80 °C until analysis. TG, TCH, HDL-c, and FSG were measured by using enzymatic and colorimetric methods according to the kits’ instructions (Beijing BHKT Clinical Co.,Ltd.). ELISA was employed for the determination of FINS and serum apoA-1 (Rapidbio INC).

### Euglycemic-hyperinsulinemic clamp study

The rats fasted for a 14 h period in advance (18:00 to 8:00) of the experiment. After intramuscular anesthesia was administered, cannula of the right jugular vein and left common carotid artery was carried out. Intravenous infusion channels and three-limb tubes were established, of which one end was connected to the insulin pump, and the other end was connected to the glucose injection pump. The basal blood glucose was required to be measured continuously 3 times, with intervals of 5 min each time after the blood glucose was stable. The infusion of human insulin (Humulin R, Novo Nordisk) was conducted at a constant rate of 4 mU · kg^−1^ · min^−1^, with the blood glucose measurement time of 5 min for each interval. The rate of the glucose infusion was adjusted according to the measured blood glucose value, making sure that there was stability in the blood glucose value (basal blood glucose value ± 0.5) mmol/L. When 3 consecutive glucose values were all in the above range, the evaluation index of the insulin sensitivity of the rats was the average value of the GIR in 60 to 120 min.

### Real time quantitative PCR

10 to 20 mg rat liver tissues was collected and put into centrifuge tubes. RNA was extracted using a Qiagen RNeasy mini kit and reverse transcribed to cDNA using TAKARA RT reagent kit for production of a template for real time qPCR. A total RNA of 1 μg was added to the reverse transcription system of 20 μL for reaction at 37 °C for 15 min, and 85 °C for 5 seconds. Real-time qPCRs were performed with an IQ5 instrument and software mainly as follows: 20 μl reaction system; 95 °C for 30 s for the pre-degeneration; after which 95 °C for 5 s for the denaturation; and annealing and extension of 30 s for 40 cycles. A standard curve was generated for all genes studied using serial dilutions of the primers specified PCR products. Melt curve generation were performed to determine the purity of the amplified PCR products. There were three repetition sets for each sample and each gene was expressed as number of copies relative to those measured for β-actin within the same RNA sample. Primers and their sequences are summarized in Table 3 (Shanghai Ying Wei Jie Ji Trading Co. Ltd.).

**Table 3:** Primers for analysis of transcription level of gene expression

Gene	Accession no.	Primer	Tm, °C	Product length,bp
ABCA1	NM_178095.2	F 5′-AGAGCTAGGTCTCCCTT-3′	56	151
		R 5′-CACTGCCCCTGTAATGG-3′		
apoA-1	NM_012738	F 5′-GAACAAGGACCTGGAGAATG-3′	58	315
		R 5′-CTGGCCTTGGTATGATACTC-3′		
actin	NM_031144.2	F 5′-CCCATCTATGAGGGTTACGC-3′	50	150
		R 5′-TTTAATGTCACGCACGATTTC-3′		

### Western-blotting detection

The liver tissues were collected and frozen in liquid nitrogen immediately until homogenization. Then, 14,000 × g, at 4 °C for centrifugation for 10 min. The concentration of protein in the supernatant was measured using a BCA protein assay kit (Beyotime, China). A 10 % sodium dodecyl sulfate (SDS)-polyacrylamide gels electrophoresis experiment was then conducted, under the conditions of the equal amount of total proteins of the 3 groups of CHF, PO and FO. The total protein was transferred onto a polyvinylidene difluoride (PVDF) membrane and incubated overnight with 5 % (w/v) nonfat dried milk, and then probed overnight with primary antibodies against β-actin, ABCA1 (all antibodies were purchased from Abcam Inc.,USA) at 4 °C. After washing the membrane with TBST, the membrane was incubated with the secondary antibody for 1 h at room temperature. Proteins were visualized using an enhanced chemiluminescence (ECL) kit in accordance with the manufacturer’s instructions. The β-actin western blot was performed as an internal control of protein loading.

### Statistical analysis

The experimental data was expressed as a mean ± standard deviation ($\overline{x}\pm s$). Data was entered into a database and analyzed using SAS9.0 software, Student’s t-test, or one-way ANOVA. Differences were considered to be statistically significant at *p* < 0.05.

## Abbreviations

ABCA1: adenosine triphosphate (ATP) binding cassette transporter A1
ALA: a-linolenic acid
apoA-1: apolipoprotein A-1
CHF: continuous high fat
DHA: docosahexaenoic acid
ECL: chemiluminescence
EPA: eicosapentaenoic acid
FDA: Food&Drug Administration
FINS: fasting insulin
FO: fish oil
FSG: fasting serum glucose
GIR: glucose infusion rate
HDL-c: high density lipoprotein cholesterol
HF: high fat
IR: Insulin resistance
MetS: metabolic syndrome
NC: normal control
PO: perilla oil
PUFA: polyunsaturated fatty acids
PVDF: polyvinylidene difluoride
RCT: reverse cholesterol transport
SD: Sprague–Dawley
SDS: sodium dodecyl sulfate
SPF: specific pathogen-free
TCH: total cholesterol
TG: triglyceride
LXRs: Liver X receptors

## References

[1] Yin J, Li M, Xu L (2013). Insulin resistance determined by Homeostasis Model Assessment (HOMA) and associations with metabolic syndrome among Chinese children and teenagers[J]. Diabetology Metabolic Syndrome.

[2] Castro AVB, Kolka CM, Kim SP (2014). Obesity, insulin resistance and comorbidities? Mechanisms of association[J]. Arquivos Brasileiros de Endocrinologia Metabologia.

[3] Ho M, Garnett SP, Baur LA (2014). Childhood obesity and insulin resistance: how should it be managed?[J]. Current Treat Options Cardiovascular Med.

[4] Marcovecchio ML, Mohn A, Chiarelli F (2010). Obesity and insulin resistance in children[J]. J Pediatr Gastroenterol Nutr.

[5] Kim S, Sohn I, Ahn JI (2004). Hepatic gene expression profiles in a long-term high-fat diet-induced obesity mouse model[J]. Gene.

[6] Hanke D, Zahradka P, Mohankumar SK, et al. A diet high in α-linolenic acid and monounsaturated fatty acids attenuates hepatic steatosis and alters hepatic phospholipid fatty acid profile in diet-induced obese rats [J]. Prostaglandins, Leukotrienes and Essential Fatty Acids (PLEFA), 2013, 89(6): 391–401. 10.1016/j.plefa.2013.09.009 24140006

[7] Zhang T, Zhao S, Li W (2014). High-fat diet from perilla oil induces insulin resistance despite lower serum lipids and increases hepatic fatty acid oxidation in rats[J]. Lipids Health Dis.

[8] Zhang Y, Liu J, Yao J (2014). Obesity: Pathophysiology and Intervention[J]. Nutrients.

[9] Kissebah AH, Freedman DS, Peiris AN (1989). Health risks of obesity[J]. Med Clin North Am.

[10] Zhao B, Wall RJ, Yang J (2005). Transgenic expression of myostatin propeptide prevents diet-induced obesity and insulin resistance[J]. Biochem Biophys Res Commun.

[11] Lewis GF, Rader DJ (2005). New insights into the regulation of HDL metabolism and reverse cholesterol transport. CircRes.

[12] Fielding CJ, Fielding PE (1995). Molecular physiology of reverse cholesterol transport. J Lipid Res.

[13] Moller DE (2001). New drug targets for type 2 diabetes and the metabolic syndrome[J]. Nature.

[14] Garg ML, Blake RJ, Clayton E (2007). Consumption of an n-3 polyunsaturated fatty acid-enriched dip modulates plasma lipid profile in subjects with diabetes type II[J]. Eur J Clin Nutr.

[15] Anderson BM, Ma DW (2009). Are all n-3 polyunsaturated fatty acids created equal[J]. Lipids Health Dis.

[16] Zhao YT, Chen Q, Sun YX (2009). Prevention of sudden cardiac death with omega-3 fatty acids in patients with coronary heart disease: a meta-analysis of randomized controlled trials[J]. Ann Med.

[17] Anghelescu I (2010). Omega-3 fatty acids for CHD with depression[J]. JAMA.

[18] Kusunoki C, Yang L, Yoshizaki T (2013). Omega-3 polyunsaturated fatty acid has an anti-oxidant effect via the Nrf-2/HO-1 pathway in 3 T3-L1 adipocytes[J]. Biochem Biophys Res Commun.

[19] Guelzim N, Huneau JF, Mathé V, et al. N-3 fatty acids improve body composition and insulin sensitivity during energy restriction in the rat [J]. Prostaglandins, Leukotrienes and Essential Fatty Acids (PLEFA), 2014, 91(5): 203–211. 10.1016/j.plefa.2014.07.007 25172359

[20] Hoofnagle AN, Vaisar T, Mitra P (2010). HDL lipids and insulin resistance[J]. Curr Diab Rep.

[21] Harris WS (1997). n-3 Fatty Acids and Serum Lipoproteins: Human Studies. Am J Clin Nutr.

[22] le Morvan V, Dumon MF, Palos-Pinto A (2002). n-3 FA increase liver uptake of HDL-cholesterol in mice[J]. Lipids.

[23] Kuang YL, Eric Paulson K, Lichtenstein AH (2012). Regulation of the expression of key genes involved in HDL metabolism by unsaturated fatty acids[J]. Br J Nutr.

[24] Oram JF, Lawn RM (2001). ABCA1: the gatekeeper for eliminating excess tissue cholesterol[J]. J Lipid Res.

[25] Li J, Fan LM, George VT, Brooks G (2007). Nox2 regulates endothelial cell cycle arrest and apoptosis via p21^cip1^ and p53. Free Radic Biol Med.

[26] Young JE, Martinez RA, La Spada AR (2009). Nutrient deprivation induces neuronal autophagy and implicates reduced insulin signaling in neuroprotective autophagy activation [J]. J Biol Chem.

[27] Shah PK, Kaul S, Nilsson J (2001). Exploiting the vascular protective effects of high-density lipoprotein and its apolipoproteins: an idea whose time for testing is coming, part II. Circulation.

[28] Lewis GF, Rader DJ (2005). New insights into the regulation of HDL metabolism and reverse cholesterol transport[J]. Circ Res.

[29] Zhang YZ, Zanotti I, Reilly MP (2003). Overexpression of apolipoprotein AI promotes reverse transport of cholesterol from macrophages to feces in vivo[J]. Circulation.

[30] Lluís L, Taltavull N, Muñoz-Cortés M (2013). Protective effect of the omega-3 polyunsaturated fatty acids: Eicosapentaenoic acid/Docosahexaenoic acid 1: 1 ratio on cardiovascular disease risk markers in rats[J]. Lipids Health Dis.

[31] Joyce C, Freeman L, Brewer HB (2003). Study of ABCA1 function in transgenic mice[J]. Arterioscler Thromb Vasc Biol.

[32] Singaraja RR, Fievet C, Castro G (2002). Increased ABCA1 activity protects against atherosclerosis[J]. J Clinical Investig.

[33] Uehara Y, Miura S, Eckardstein A (2007). Unsaturated fatty acids suppress the expression of the ATP-binding cassette transporter G1(ABCG1) and (ABCA1) genes via an LXR/RXR responsive element. Atherosclerosis.

[34] Salehipour M, Javadi E, Reza JZ (2010). Polyunsaturated fatty acids and modulation of cholesterol homeostasis in THP-1 macrophage-derived foam cells[J]. Int J Mol Sci.

[35] Zhang L, Geng Y, Xiao N (2009). High dietary n-6/n-3 PUFA ratio promotes HDL cholesterol level, but does not suppress atherogenesis in apolipoprotein E-null mice 1[J]. J Atheroscler Thromb.

